# A Unique Case of Nonhypoxic Splenic Infarction in a Patient With Sickle Cell Trait Due to Dehydration and Sepsis From a Dental Infection: A Case Report

**DOI:** 10.7759/cureus.26645

**Published:** 2022-07-07

**Authors:** Saira Chaughtai, Waqar Akram, Khaula Chaughtai, Zeeshan Chaughtai, Arif Asif

**Affiliations:** 1 Internal Medicine, Jersey Shore University Medical Center, Neptune, USA

**Keywords:** sepsis, dehydration, dental infection, splenic infarction, sickle cell trait

## Abstract

Splenic infarction is a recognized complication occurring at high altitudes in patients with sickle cell trait (SCT). There have been a few cases of splenic infarction occurring in the setting of nonhypoxic events at sea level, and some cases of spontaneous splenic infarction without an inciting event in patients with SCT.

We present the case of a 25-year-old male with a recent untreated dental infection who was drinking alcohol excessively and moving furniture over the last few weeks. He came to the hospital due to left-sided abdominal pain and was found to have a splenic infarction. He was found to have hemolysis on his blood work, with elevated lactate dehydrogenase, low haptoglobin, and elevated bilirubin levels. He underwent hemoglobin electrophoresis which revealed SCT. His blood cultures grew *Streptococcus mitis* and *Streptococcus oralis*, a normal oral commensal, which was thought to be due to the untreated dental infection. His workup for endocarditis as a source of splenic infarction was negative, and he had no other source of emboli. He was treated with antibiotics for the sepsis and fluids for the sickling and hemolysis. He developed multiple complications of the splenic infarction but ultimately recovered.

Ours is the first example of nonhypoxic splenic infarction in an SCT patient that has been documented in a scenario of dehydration and sepsis. This link should be understood to prevent splenic infarction even at sea level by preventing overexertion and dehydration in individuals with known SCT.

## Introduction

The heterozygous state of the sickle hemoglobin beta-globin gene (HbAS) is carried by as many as 300 million individuals and up to 40% of the population in some regions of the world [1]. Its main prevalence is in Africa, Asia, and the Middle East [1]. It is also found in the United States due to the diversity of the population. Sickle cell trait (SCT) has an evolutionary advantage as it has a protective effect against malaria [1]. However, there are potential serious morbidities in SCT individuals, including increased incidence of renal failure, venous thromboembolism, splenic infarction as a high-altitude complication, and exercise-related rhabdomyolysis and sudden death [1]. There have been few cases of splenic infarction occurring in patients at sea level. Here, we review the literature regarding splenic infarction in SCT patients and present a unique case associated with dehydration and sepsis occurring at sea level.

## Case presentation

A 25-year-old man having both African and Caucasian heritage presented to the hospital with a one-day history of left-sided abdominal pain as his only complaint. For the prior two weeks, he had been binging alcohol and moving furniture into a new apartment. Two weeks ago, he had cracked a tooth but had not sought dental attention. His medical history was significant for asthma. He took no medications. His paternal uncle has sickle cell disease.

His vitals were normal, with a blood pressure of 105/80 mmHg, heart rate of 76 beats per minute, respiratory rate of 15 per minute, and temperature of 98.9°F, with an oxygen saturation of 95%. An abdominal examination revealed tenderness in the left upper and middle quadrants with no organomegaly.

**Figure 1:**
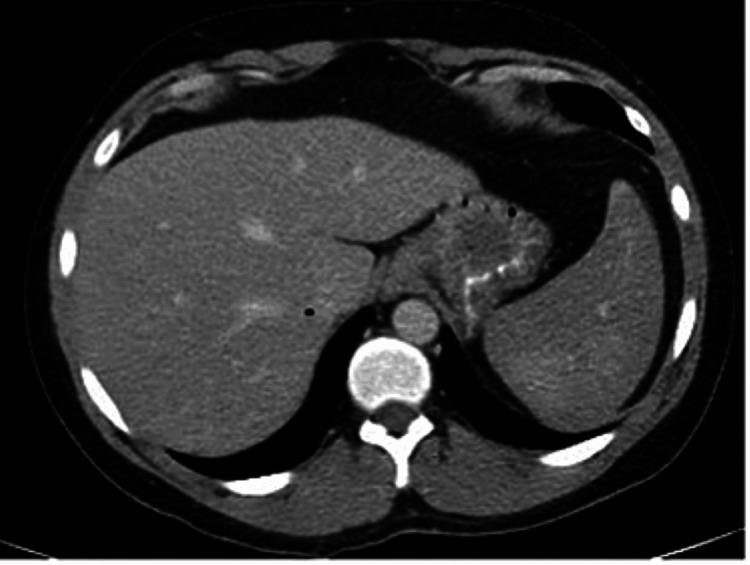
Initial computed tomography of the abdomen and pelvis showing splenic enhancement on day one.

**Figure 2:**
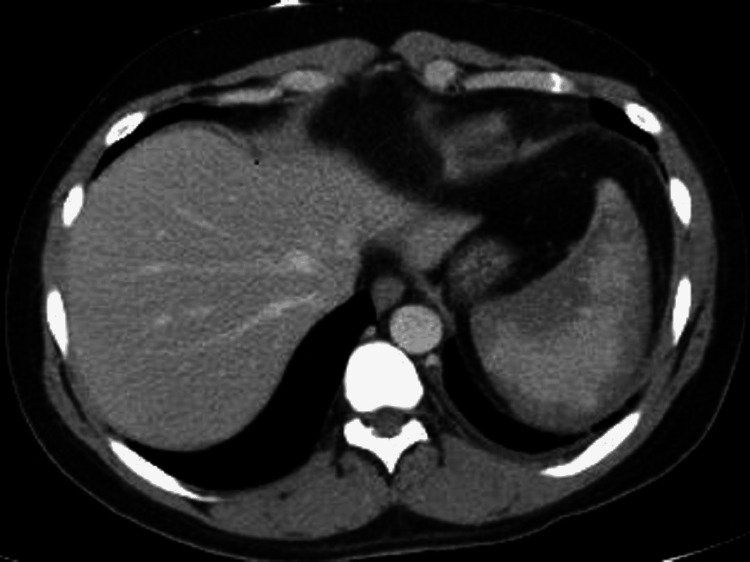
Subsequent computed tomography of the abdomen and pelvis showing the progression of splenic enhancement on day two.

Initial laboratory studies showed elevated white blood cell count (WBC) of 16,000/µL (normal: 4,500-11,000/µL), normal hemoglobin 14 g/dL (normal: 13-17 g/dL), and low platelets of 117,000/µL (normal: 140,000-450,000/µL) (Table 1). Lactate dehydrogenase (LDH) was elevated at 1,562 IU/L (normal: 91-200 IU/L), haptoglobin was low at 10 mg/dL (normal: 30-225 mg/dL), total bilirubin was elevated at 2 mg/dL (normal: <1.1 mg/dL), and direct bilirubin was elevated at 0.3 mg/dL (normal: <0.3 mg/dL), indicating a hemolytic process. Creatine phosphokinase (CPK) was high at 514 IU/L (normal: 22-232 IU/L). Mono-spot test was negative. Computed tomography (CT) of the abdomen and pelvis with contrast showed splenic enhancement (Figure 1). Repeat imaging the next day for worsening abdominal pain radiating to the left shoulder revealed perisplenic fluid, trace left pleural effusion, and possible splenic infarction (Figure 2).

**Table 1:** Initial laboratory findings of the patient.

Laboratory test	Result	Reference range
White blood cell	16,000/µL	4,500–11,000/µL
Hemoglobin	14 g/dL	13–17 g/dL
Platelet	117,000/µL	140,000–450,000/µL
Lactate dehydrogenase	1,562 IU/L	91–200 IU/L
Haptoglobin	10 mg/dL	30–225 mg/dL
Total bilirubin	2 mg/dL	<1.1 mg/dL
Direct bilirubin	0.3 mg/dL	<0.3 mg/dL
Creatine phosphokinase	514 IU/L	22–232 IU/L

Admission blood cultures grew *Streptococcus mitis* and *Streptococcus oralis*, a normal oral commensal. It was believed that the sepsis was caused by the tooth that he had shattered, as well as the dental infection that he had not sought medical assistance for. Ceftriaxone was initiated, and evaluation for endocarditis as a cause of septic emboli and splenic infarction ensued. Transthoracic echocardiogram (TTE) and transesophageal echocardiogram (TEE) did not show any vegetations. Further questioning revealed that he had sickle cell disease on one side of his family. A sickle screen was positive, and electrophoresis showed hemoglobin A1 of 55% (normal: 96-100%), hemoglobin S of 41.8% (normal: 0%), and hemoglobin A2 of 3.2% (normal: 0-4%), which was compatible with SCT. His splenic infarction was attributed to SCT.

A hypercoagulable workup, including levels of beta 2 glycoprotein, antithrombin III, protein C, anticardiolipin, and JAK2 mutation, was all negative. A rheumatologic workup including levels of antineutrophil cytoplasmic antibodies, antinuclear antigen, and rheumatoid factor was negative. His hemoglobin dropped from 14 to 11 g/dL during the ensuing days. He was given hydration and analgesia.

**Figure 3:**
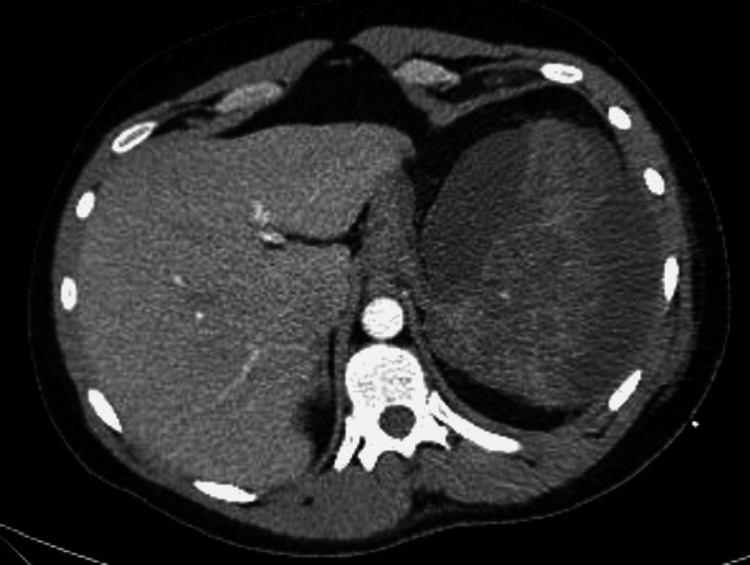
Subsequent computed tomography of the abdomen and pelvis showing the progression of splenic infarction with significant perisplenic fluid on day nine.

**Figure 4:**
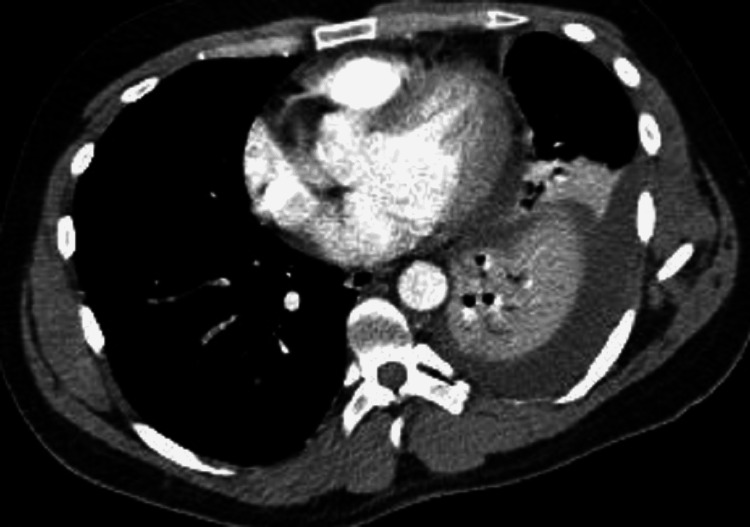
Subsequent computed tomography of the abdomen and pelvis showing left pleural effusion and compressive atelectasis on day nine.

The hospital course was complicated by perisplenic fluid and ascites (Figure 3), for which he had a peritoneal tube that was placed and then removed. He also had recurrent left pleural effusion (Figure 4), for which he had chest tube drainage twice. He was seen by cardiothoracic surgery for a loculated left pleural effusion, for which he underwent left-sided video-assisted thoracoscopic surgery and decortication. All cultures from the pleural and peritoneal drains were negative. He completed two weeks of ceftriaxone 2 g daily.

The pain eventually subsided, and the hemoglobin stabilized. Splenectomy was not performed to avoid future immune deficiency. The patient was advised to avoid overexertion and volume depletion in the future.

## Discussion

The heterozygous state of sickle HbAS is carried by as many as 300 million individuals globally, with the largest prevalence in Africa, Asia, and the Middle East [1]. SCT has an evolutionary advantage in protecting from malaria [1]. However, it has been associated with various complications, including venous thromboembolism and kidney disease [1].

In the United States, recent focus has been on the relevance of screening military and college athlete recruits for SCT [1]. The reason for this is the increased incidence of exercise-induced rhabdomyolysis and sudden death in SCT patients who are athletes, with a 37-fold higher risk of the latter [2]. There has even been the question of whether to screen athletes for SCT [3]. It was proposed by Shephard that it may be more cost-effective to advise hydration and avoid overexertion for all athletes [3].

It should be recognized that SCT is not just an occurrence in the black population. The study conducted by the Centers for Disease Control and Prevention reports an estimated incidence of 73.1 cases per 1,000 black newborns, 6.9 cases per 1,000 Hispanic newborns, 3.0 cases per 1,000 Caucasian newborns, and 2.2 cases per 1,000 Asian/Pacific Islander newborns [4].

There have been definite associations of splenic infarction occurring from sickling in SCT patients who ascend to higher altitudes. Hypoxia at higher altitudes induces sickling of red blood cells in those with SCT [5]. Franklin et al. describe four such patients, two of whom were black, one was Hispanic, and one was Caucasian [5].

In our case report, we described a patient who was drinking alcohol excessively and exerting himself by moving furniture. When he was admitted to the hospital with left abdominal pain, he was found to have a new splenic infarction that evolved over the hospital stay. His laboratory values showed elevated WBC and LDH and a drop in hemoglobin. He was found to have sepsis from a recent tooth infection. Given that extensive workup for an embolic source was not found with TTE and TEE, his splenic infarction was attributed to dehydration and sepsis. Based on our review, this is the first reported case of splenic infarction occurring at a low altitude due to dehydration and sepsis in an SCT patient.

Splenic infarction has been shown to not always occur in SCT patients in the setting of hypoxia [6]. Gitlin et al. reported six cases of splenic sequestration that were not related to altitudes that clinically presented similarly to our patient [7]. All patients presented with abdominal pain with left-sided pleural effusion, an increase in WBC and LDH, and a sudden drop of hemoglobin, with distribution between hemoglobin S and hemoglobin A ranging from 25-40% and 55-75%, respectively [7].

Baron et al. described a case of bacterial endocarditis in a patient with SCT causing splenic rupture [8]. In our patient’s case, he did not have evidence of endocarditis or any other embolic source. His splenic infarction was attributed to dehydration-related sickling and sepsis. There was also a report of sickle cell thrombi occurring after open-heart surgery in a patient with SCT [9].

There was even a case report of a patient with SCT who developed splenic infarction without any predisposing factor [10]. Seegars et al. discussed a review of 12 patients who developed nonhypoxic splenic infarction, five of whom were spontaneous and without any inciting event [11].

Other conditions causing nonhypoxic splenic infarction in SCT patients have been described as well. Asfaw et al. published a case of cocaine crisis causing splenic infarction and multiple other organ infarctions in a patient with SCT [12]. There have also been cases of splenic infarction in SCT patients associated with Epstein-Barr virus (EBV) [13,14]. An interesting case of hypoxic pulmonary embolism inducing splenic infarction occurred in a patient three weeks after abdominal surgery [15].

Patients with SCT are known to develop sickling and splenic infarction at higher altitudes. At sea level, some of the main morbidities of SCT are kidney disease, exercise-induced rhabdomyolysis that causes sickling, venous thromboembolism, and sudden death. There have been various reports of SCT patients developing splenic infarction at low altitudes. These cases have been found to be due to infections including EBV, drugs like cocaine, hypoxia-inducing pulmonary embolism, thromboembolic from endocarditis, and after open-heart surgery. Ours is the first such case of splenic infarction occurring in the setting of dehydration from alcohol intake and physical exertion of moving furniture. Moreover, our patient had sepsis from a tooth infection. The combination of dehydration and sepsis was enough to induce splenic infarction in our patient.

## Conclusions

Splenic infarction is a recognized complication occurring at high altitudes in patients with SCT. There have been a few cases of splenic infarction occurring in the setting of nonhypoxic events at sea level, and some cases of spontaneous splenic infarction with no inciting event in patients with SCT. Ours is the first example of nonhypoxic splenic infarction occurring in an SCT patient that has been documented in a scenario of dehydration and sepsis. This relationship must be understood to prevent splenic infarction in individuals with known SCT even at sea level.
